# Formation of supported lipid bilayers of charged *E. coli* lipids on modified gold by vesicle fusion

**DOI:** 10.1016/j.mex.2017.11.002

**Published:** 2017-11-14

**Authors:** Ileana F. Márquez, Marisela Vélez

**Affiliations:** Instituto de Catálisis y Petroleoquímica-CSIC, c/Marie Curie 2, Cantoblanco, Madrid 28049, Spain

## Abstract

We describe a simple way of fusing *E. coli* lipid vesicles onto a gold surface. Supported lipid bilayers on metal surfaces are interesting for several reasons: transducing a biological signal to an electric readout, using surface analytical tools such as Surface Plasmon Resonance (SPR), Infrared Reflection Absorption Spectroscopy, Neutron Reflectivity or Electrochemistry. The most widely used method to prepare supported lipid membranes is fusion of preexisting liposomes. It is quite efficient on hydrophilic surfaces such as glass, mica or SiO_2_, but vesicle fusion on metals and metal oxide surfaces (as gold, titanium oxide or indium tin oxide), remains a challenge, particularly for vesicles containing charged lipids, as is the case of bacterial lipids. We describe a simple method based on modifying the gold surface with a charged mercaptopropionic acid self-assembled monolayer and liposomes partially solubilized with detergent. The formed bilayers were characterized using a Quartz Crystal Microbalance with dissipation (QCM-D) and Atomic Force Microscopy (AFM). Some advantages of this protocol are that the stability of the self-assembled monolayer allows for repeated use of the substrate after detergent removal of the bilayer and that the amount of detergent required for optimal fusion can be determined previously using the lipid-detergent solubility curve.

## Graphical abstract

**Figure:**
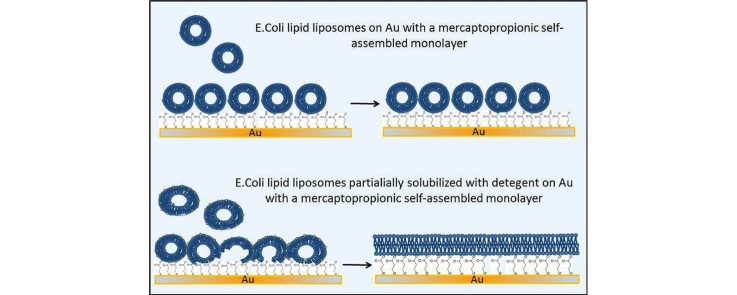

## Method details

### Materials

• Chemicals used (≥99.0% purity) were purchased from Sigma-Aldrich (Madrid, Spain).
• Lyophilized l-α-phosphatidylcholine (egg yolk, EPC), *E. coli* polar lipid extract (EcPl), l-α-phosphatidic acid (EPA) and 1,2-dioleoyl-*sn*-glycero-3-[(*N*-(5-amino-1-carboxypentyl)iminodiacetic acid)succinyl] (DOGS-NTA) nickel salt were purchased at Avanti Polar Lipids, Inc. (Alabaster, AL).

*Note:* lyophilized lipids were stored at −20 °C until used. Lipid stock solution were prepared by dissolving the lipid powder in a mixture of chloroform:methanol (1:1, v/v) at 10 mg/mL and stored at −20 °C until used.

• 4–Aminothiophenol and 3–mercaptopropionic acid were purchased from Sigma-Aldrich (Madrid, Spain).
• *N*-Dodecyl β-d-maltoside surfactant was purchased from Sigma-Aldrich (Madrid, Spain).
• Bio-beads (^®^Carbosorb).
• Atomic Force Microspcopy (AFM) images were acquired with a microscope from Nanotec Electrónica (Madrid, Spain) operated in the jump mode [1].

*Note:* Measurements were always performed under liquid conditions in standard buffer at room temperature using silicon nitride cantilevers (Olympus, RC800PSA) with a spring constant of 0.05 N/m, and an estimated tip radius of 20 nm. All images were first-order flattened using WSxM software from Nanotec Electrónica [2].

• Gold on glass substrates 11 × 11 mm size were purchased from Arrandee™, Germany.

*Note*: *Au*-coated substrates (1 × 1 cm^2^) consist of 200 nm gold over 14 nm of chromium prepared on borosilicate glass and were used for the AFM studies.

• Quartz crystal microbalance with dissipation (QCM-D) measurements were performed using a QCM-Z500 from KSV (Finland) resonator with dissipation monitoring.

*Note:* The sample was continuously delivered to the measurement chamber by a syringe pump (^kd^Scientific, model KDS120) at a constant flow rate of 50 μL/min. Temperature was internally controlled by a peltier and externally by a Nahita refrigerated thermoblock (model 603/20), and all measurements were performed at 23 ± 0.5 °C.

• QCM *SiO_2_* and *Au* coated 5 MHz quartz crystals were purchased at Q-Sense.
• UV/ozone cleaner (Bioforce Nanoscience, model UV.TC:220) was used to clean the *SiO_2_* QCM substrates.
• Liposome integrity was followed in a Spectrophotometer UV-2401 PC (UV–vis recording spectrophotometer, SHIMADZU) by recording the absorbance at 350 nm wavelength while adding desired amounts of the surfactant.

## Procedure

*The formation of a supported lipid bilayer (SLB) on a metal surface can be shortly described in the following steps: liposome preparation, partial solubilization with detergent, surface cleaning, formation of a self-assembled monolayer (SAM) on gold substrates, incubation of solubilized liposomes on the modified gold to form the SLB*

1. Standard buffers used were 10 mM Tris-HCl, 200 mM NaCl adjusted to pH 7.5 with NaOH, and Na_2_HPO_4_ 0.1 M adjusted to pH 5.5, both buffer solutions with or without 2 mM CaCl_2_.
  *Note:* All buffer solutions were prepared with distilled and deionized water Milli-Q (Millipore^®^) and were filtered through a 0.2 μm pore-diameter cellulose syringe filter immediately before use. For QCM experiments, buffers were degassed 1 h at room temperature previous to use with an ultrasonic cleaner Bransonic^®^ (B-2510 model) operated in the degassing mode.
2. Liposomes were prepared by mixing a desired amount of lipid stock solution in a round bottomed flask and gently dried under nitrogen stream for 30 min. The dried lipid film was hydrated in an appropriate buffer vortexing for 30 min until having a homogeneous mixture. The resulting multilamellar vesicle (MLV) suspensions were run through an Avanti mini extruder (Alabaster, AL) with a polycarbonate membrane of known pore size (50 or 200 nm) to form large unilamellar vesicles (LUVs). Liposomes solutions were immediately stored in airtight containers at 4 °C for no more than one week. Storing liposomes for this amount of time did not affect the experiments for the formation of a supported lipid bilayer.
3. The integrity of the liposomes, was checked by performing a solubility curve to determine the right amount of the detergent DDM needed for partial destabilization.
  *Note:* The turbidity of a liposome solution at increasing detergent concentrations is followed spectrophotometrically observing the absorbance at 350 nm as previously described on membrane protein reconstitution protocols [3]. Fig. S1 shows a typical liposome-detergent solubility curve. When the absorbance was below 60% of the initial value, the partially dissolved vesicles are able to fuse on the gold surface.
4. AFM gold substrates were cleaned with piranha solution (3:1 H_2_SO_4_ 98%/H_2_O_2_ 30%) and rinsed extensively with Milli-Q water. *(Caution! Piranha solution is especially dangerous and corrosive and may explode if contained in a closed vessel. It should be handled with special care.).* The substrates were then annealed to an orange glow for a few seconds in a propane flame; this operation was repeated twelve times. This treatment is known to produce Au(111) terraces of a few micrometers’ radius with atomically flat surfaces separated by deep boundaries, suitable for AFM characterization.
5. QCM substrates were treated as follows:
  a Before each measurement *SiO_2_*-coated crystals were cleaned in a 2% (w/v) sodium dodecyl sulfate (SDS) solution for 15 min, rinsed extensively with distilled water, dried under a stream of nitrogen gas and exposed to the UV/ozone cleaner for at least 30 min. The exposure to the UV light cleans the *SiO_2_* by eliminating organic contaminants and oxidizing the silicon surface rendering it hydrophilic. After cleaning, crystals were mounted in the QCM chamber immediately.
  b *Au*–coated crystals were cleaned with a piranha solution (3:1 H_2_SO_4_ 98%/H_2_O_2_ 30%), followed by extensive rinsing with distilled Milli-Q water and dried under a nitrogen stream.
6. Clean *Au* coated surfaces were then submerged in a solution of 0.2 mM 3–mercaptopropionic acid (deionized water: ethanol, 1:1) at room temperature and left over night (18 hs) until a self-assembled monolayer (SAM) was formed. The substrates were rinsed with distilled Milli-Q water, dried under a stream of nitrogen immediately before use.
7. Although sample preparation depends on the measurement technique used, liposome solution was diluted at 0.1 mg/mL:
  a For AFM measurements a certain volume of partially solubilized liposomes was allowed to adsorb to the surface over night at room temperature in the presence of Bio-beads. Completed the incubation time, the non-adsorbed liposomes were removed by rinsing the sample with buffer prior to measurements.
    *Note*: A schematic representation of the liposomes adsorption is showed in Fig. S3.
  b QCM measurements start obtaining a baseline by injecting buffer into the chamber. Then, a temperature-equilibrated solution of partially solubilized liposomes was introduced into the measurement chamber. Following the liposome adsorption, the excess of non-adsorbed liposomes were removed from the chamber by rinsing with buffer.
    *Note:* Results are reported through the normalized resonance frequency f_n_/n and dissipation D_n_. The data obtained on the seventh overtone are presented in the figures. Before and after each measurement, the flow module was dismounted and extensively cleaned with distilled Milli-Q water and dried under a stream of nitrogen.

## Method validation

**Fig. 1:**
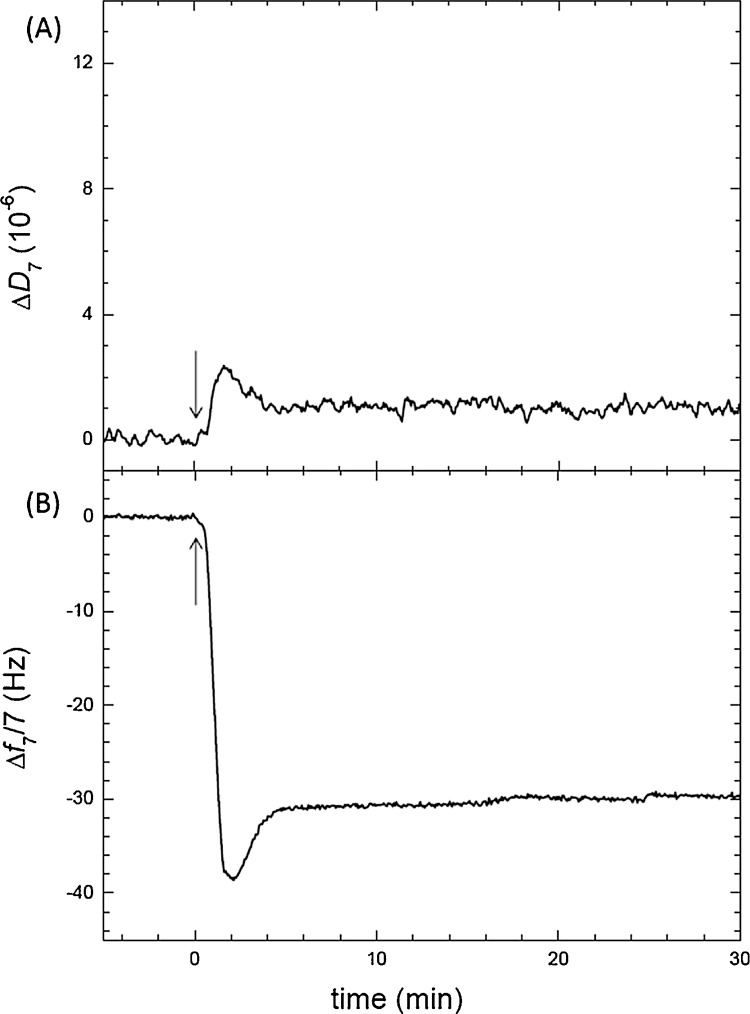
*E. coli* lipid vesicles solubilized with detergent on Au (A) represents dissipation and (B) frequency changes after liposome injection (arrow). 100% EcPl lipids solubilized with DDM as described in the text fuse to form a bilayer in the presence of 2 mM CaCl_2._

Quartz Crystal Microbalance with dissipation (QCM-D) can follow in real time vesicle fusion on gold [4] and silicon oxide surfaces and can distinguish between the adsorption of intact vesicles and the formation of a supported lipid bilayer [5], [6]. It also allows quantifying the amount of lipid deposited, detecting therefore the degree of surface coverage and efficiency of vesicle fusion. Fig. 1 shows the frequency and dissipation values observed for the detergent solubilized EcPl lipids in the presence of Ca^2+^, Δf_7_/7 = 30 ± 0.5 Hz and ΔD = (1 ± 0.5) × 10^−6^, that are within the values reported for the formation of a lipid bilayer [6]. The stability after extensive rinsing indicates the presence of a bilayer on the gold surface.

We tested this strategy to fuse liposomes containing other negatively charged lipids. PC liposomes containing 10% of EPA (negatively charged), 90% EPC (zwitterionic) molar ratio were solubilized with the same detergent. Fig. S1 shows that liposomes of this composition aggregated at low detergent concentrations. The detergent concentration used for EcPl was therefore not enough to induce vesicle fusion of EPC:EPA liposomes. However increasing the amount of detergent until the absorbance was below 60% its initial value was enough to obtain vesicle fusion. The QCM chamber permits for extensive rinsing of the surface once the bilayer is formed, allowing for detergent removal by dilution. The frequency and dissipation values obtained after fusion of liposomes containing EPC-EPA mixture are the same as the ones obtained for EcPl shown in Fig. 1.

**Fig. 2:**
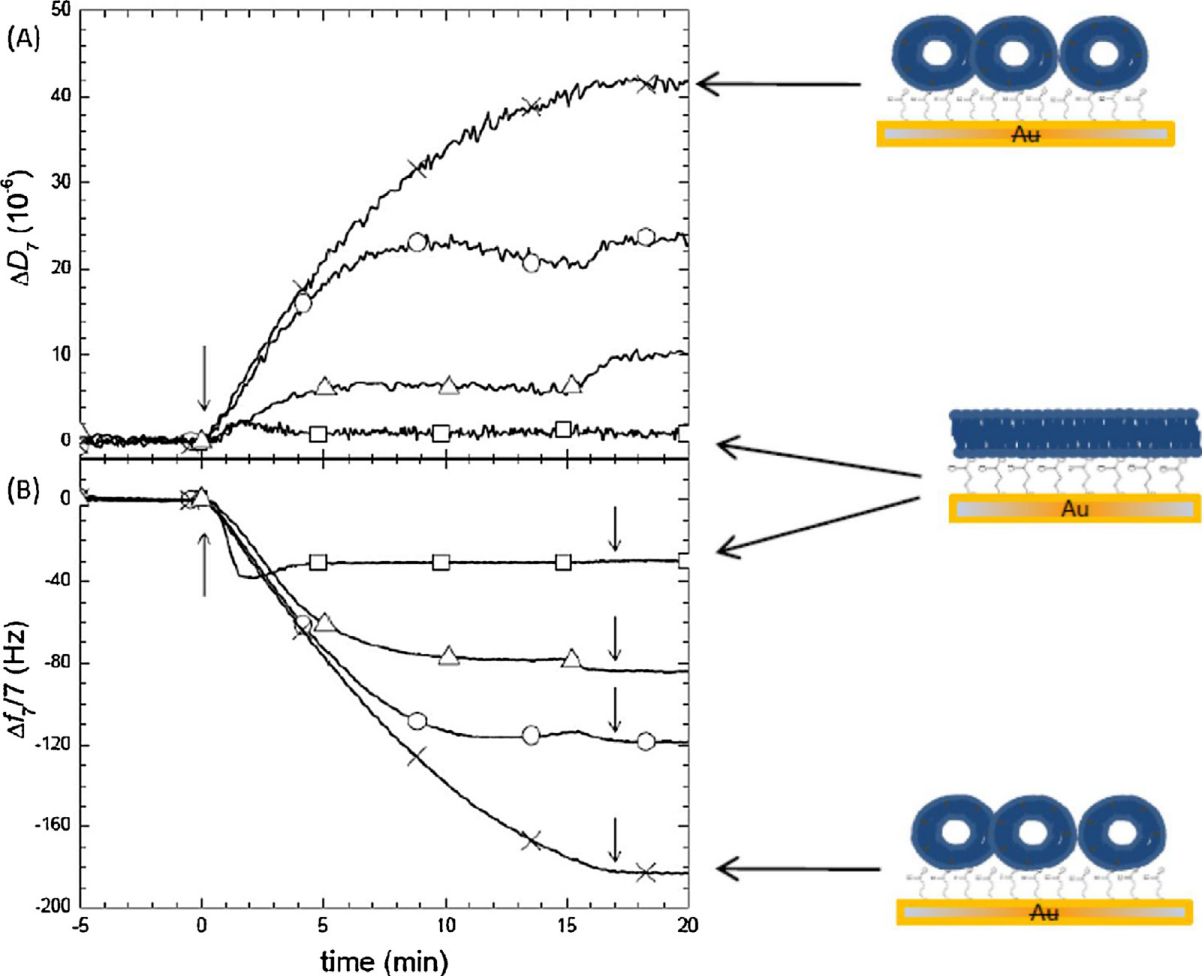
Effect of ca^2+^ Concentration on fusion of *E. coli* lipid vesicles on mercaptopropionic-modified gold. (A) represents dissipation and (B) frequency changes (—Χ—) Absence of Ca^2+^: vesicles adsorb but do not fuse (high frequency dissipation) (—○—) 0.5 mM Ca^2+^: partial vesicle fusion (—□—) 2 mMCa^2+^: complete vesicle fusion (increase frequency, low dissipation). (—Δ—) 5 Mm Ca^2+^: partial vesicle fusion Arrows at time 0 indicate liposome injection and arrows at minute 17 indicate injection of rinsing solution without liposomes. The schematic drawing at the right illustrate the expected configuration of the lipids giving rise to the signals pointed by the arrows.

We observed that EcPL liposomes containing 20% DOGS-NTA did not require previous treatment with detergent and were able to fuse on gold modified with the mercaptopropionic SAM. It is likely that the perturbation in the lipid surface caused by the presence of the NTA is enough to destabilize the membranes and allow liposome rupture and fusion. We explored with these liposomes the effect of different calcium concentrations (Fig. 2). 2 mM Ca^2+^ allowed the formation of a supported bilayer, giving frequency and dissipation changes, Δf_7_/7 = 30 ± 0.5 Hz and ΔD = (1 ± 0.5) × 10^−6^ within the values reported for the formation of a lipid bilayer [6]. Lower or higher Ca^2+^ concentrations allowed vesicle adsorption, but prevented complete liposome fusion. Previous reports have shown that the presence of 5 mM CaCl_2_ increases the deformation of zwitterionic vesicles [7] facilitating their fusion. The optimal Ca^2+^ concentration to fuse these negatively charged lipids on the mercaptopropionic-modified gold was found to be lower, only 2 mM. It would be interesting to explore also how divalent cation concentration affects the deformation of negatively charged vesicles on modified gold substrates.

The mercaptopropionic monolayer on gold proved to be stable and allowed repeated formation of bilayers. The supported bilayer could be removed in the QCM-D chamber by rinsing with a 1% SDS detergent solution to regenerate the surface. SDS has been shown to be very efficient, even at ten times lower concentrations than the one used here, to completely remove supported lipid bilayers [8]. After thorough rinsing, liposomes added in a subsequent injection also fused to form a complete bilayer, as shown in Fig. S2.

**Fig. 3:**
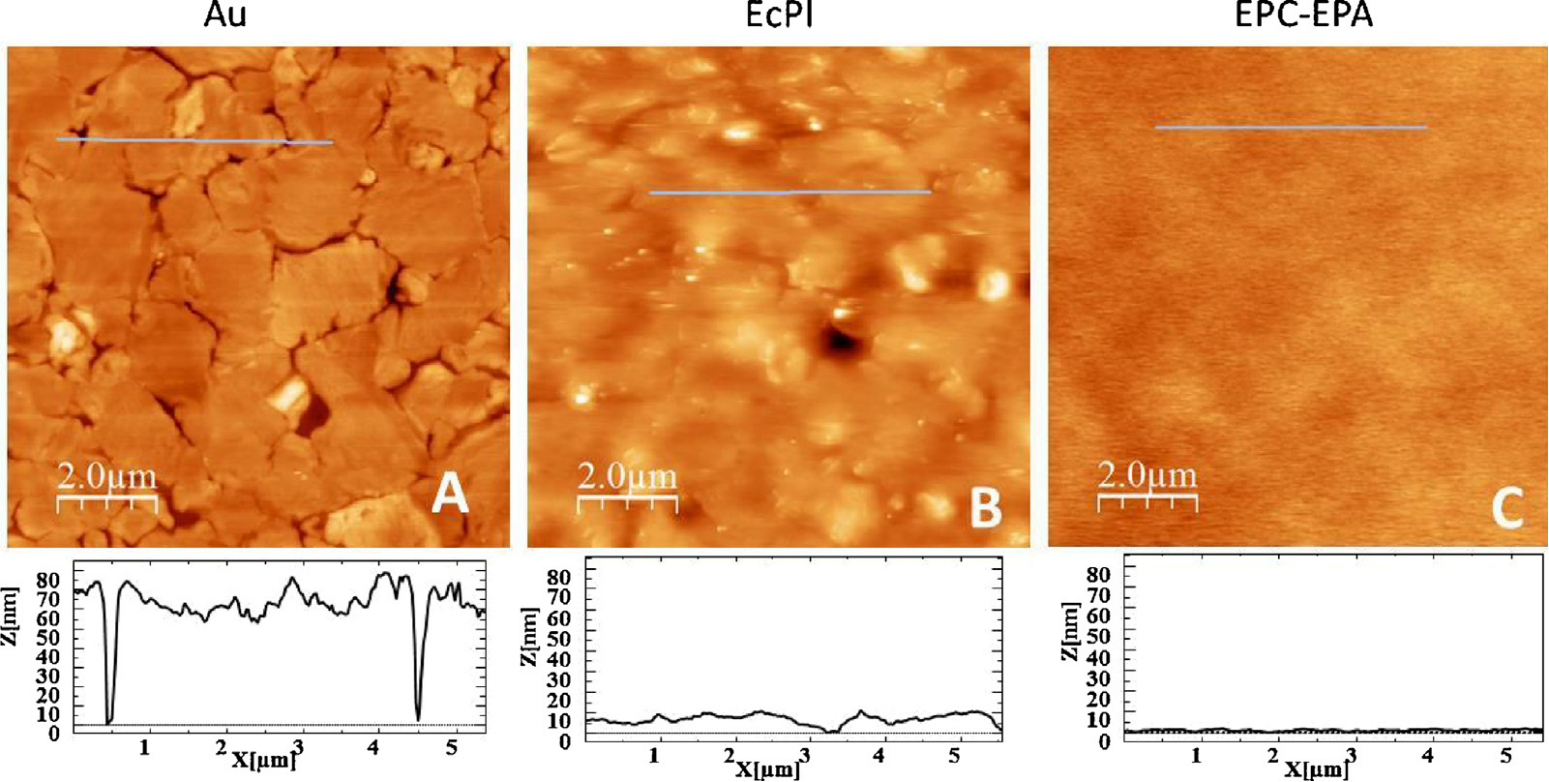
AFM images of the lipid bilayers formed on gold modified with mercaptopropionic acid (A) Image of the bare annealed gold surface (B) EcPl bilayer (C) EPC-EPA bilayer. The height profile of the blue line in the image is shown below.

In order to confirm the presence and smoothness of the bilayers formed on the mercaptopropionic-modified gold we observed the modified surfaces with Atomic Force Microscopy. Fig. 3 shows a gold substrate before and after the surface was incubated with EcPl and EPC-EPA liposomes partially solubilized with detergent. In both cases, the detergent was removed during liposome fusion by polystyrene beads (Biobeads), present during the incubation time, as is shown in the schematic drawing in Fig. S3. The height profile along the blue line shown below illustrates how the presence of the bilayer smooths the gold terraces.

## Additional information

We still lack full understanding of the process of vesicle fusion on surfaces, in spite of recent theoretical [9], [10], [11] and experimental [12], [13], [14] advances. Vesicle curvature, lateral tension, osmotic pressure, concentration and adhesion forces between the vesicles and the surface [12], [15], have been identified as relevant parameters in governing vesicle fusion. Nevertheless, tuning these parameters is not always sufficient to find conditions required to fuse *E. coli* liposomes on metal surfaces. Different strategies, such as using a viral peptide to induce vesicle fusion [16] or modifying the gold surfaces with self-assembled monolayers to modulate surface charge and hydrophobicity, have been used to facilitate vesicle fusion [17], [18], [19], [20] on metal surfaces. Partial disruption of liposomes with detergent has also been successfully used to facilitate vesicle rupture. Detergents have been used previously to assist vesicle fusion to solid [21] or polymer surfaces [22].

In the protocol presented here we combine a gold surface modified with a negatively charged self-assembled monolayers with liposomes partially solubilized with detergent to permit fusion of the negatively charged lipids on gold. It is believed that at low detergent:lipid molar ratios some detergent molecules incorporate into the liposomes, facilitating protein insertion. We found that, for EcPl, at a detergent:lipid molar ratio of about 1:3, the liposomes fused to the surface forming a bilayer. Extensive washing diluted the detergent and the structure of the membrane, as detected by AFM, are indistinguishable from other membranes prepared in the absence of detergent, supporting the interpretation that the detergent was removed upon washing.

Liposomes made from EPC:EPA (90:10 molar ratio), that is with 10% negatively charged lipids, required more detergent to solubilize and fuse. The solubility curve presented in Fig. S1 indicates that the interaction of this detergent with the liposomes is significantly different from the one observed with EcPl. At low concentrations, the turbidity increases, indicating that some lipid detergent complex could be forming and originating the formation of aggregates larger than the initial liposomes. Further detergent addition dissolves this aggregates. We found that at detergent:lipid ratios when the turbidity was below 60% of the original value the liposomes fused to the surface. It will be interesting to explore the extent to which this strategy can be extrapolated to promote disruption of vesicles of different composition on a variety of surfaces such as TiO_2_ or ITO, in which fusion of charged liposomes is challenging but highly desired.
